# Natural recovery of a marine foundation species emerges decades after landscape-scale mortality

**DOI:** 10.1038/s41598-021-86160-y

**Published:** 2021-03-26

**Authors:** Margaret O. Hall, Susan S. Bell, Bradley T. Furman, Michael J. Durako

**Affiliations:** 1 Florida Fish and Wildlife Conservation Commission, Florida Fish and Wildlife Research Institute, St. Petersburg, FL 33701 USA; 2 Department of Integrative Biology, University of South Florida, Tampa, FL 33620 USA; 3 Center for Marine Science, University of North Carolina at Wilmington, Wilmington, NC 28403 USA

**Keywords:** Ecology, Ocean sciences

## Abstract

Globally, the conditions and time scales underlying coastal ecosystem recovery following disturbance remain poorly understood, and post-disturbance examples of resilience based on long-term studies are particularly rare. Here, we documented the recovery of a marine foundation species (turtlegrass) following a hypersalinity-associated die-off in Florida Bay, USA, one of the most spatially extensive mortality events for seagrass ecosystems on record. Based upon annual sampling over two decades, foundation species recovery across the landscape was demonstrated by two ecosystem responses: the range of turtlegrass biomass met or exceeded levels present prior to the die-off, and turtlegrass regained dominance of seagrass community structure. Unlike reports for most marine taxa, recovery followed without human intervention or reduction to anthropogenic impacts. Our long-term study revealed previously uncharted resilience in subtropical seagrass landscapes but warns that future persistence of the foundation species in this iconic ecosystem will depend upon the frequency and severity of drought-associated perturbation.

## Introduction

Coastal marine ecosystems have lost resilience via decreased resistance to change or a diminished capacity to recover from disturbance, and many now appear to be on declining trajectories^1,2^. Foundation species vital to these ecosystems are increasingly challenged by human influences, including eutrophication and accelerating rates of climate change^3^. However, in some cases, ecosystem recovery has occurred even after near complete loss of foundation species, (e.g., kelp forests in the Pacific^4^ and coral reefs in western Australia^5^). Accordingly, there is heightened interest to discern both how and why some marine systems remain capable of recovery while others do not^6^.

**Figure 1:**
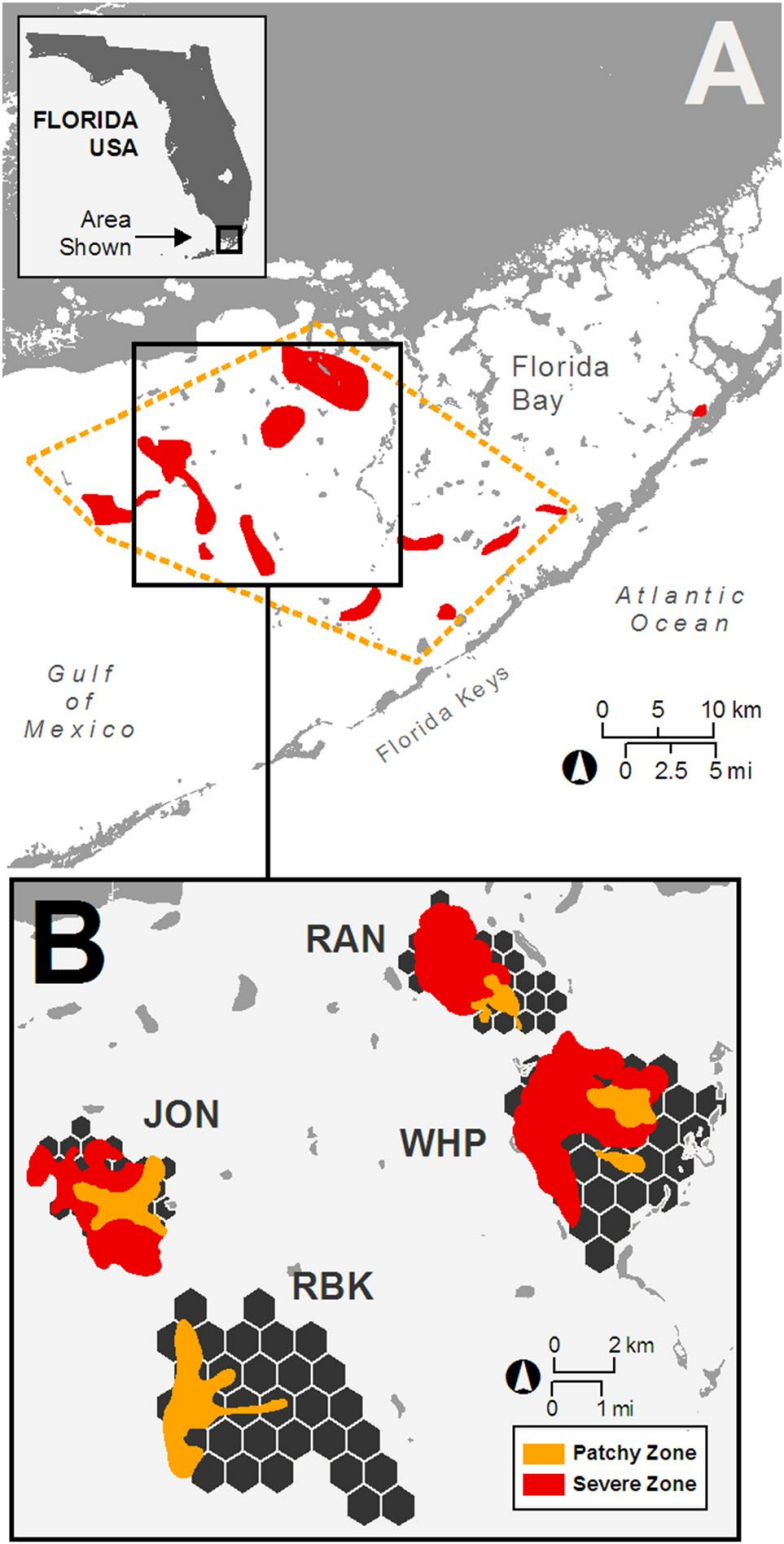
Delineation of seagrass die-off in Florida Bay, and location and die-off status of study sites. (**A**) Spatial extent of turtlegrass die-off 1987–1991 (yellow dashed line) and severely affected areas (red) as adapted from Robblee et al.^11^. (**B**) Four basins were selected as study sites (RAN: Rankin Lake, JON: Johnson Key, WHP: Whipray, and RBK: Rabbit Key). Within each basin (hexagonal areas) die-off was categorized into zones: “severe” (red), “patchy” (orange) and “unaffected” (black) based upon patterns of seagrass cover and frequency of seagrass occurrence (see Materials and Methods).

The conditions and temporal scales required to attain marine ecosystem recovery are poorly understood because studies demonstrating resilience are scarce in the literature^7^ and requisite long-term environmental and ecological records are absent for many ecosystems, particularly when documenting recovery post-disturbance^8^. Information is especially limited from pulse disturbances^9^ in tropical settings^10^. Here, the mass mortality (i.e., die-off) of *Thalassia testudinum* (turtlegrass) in Florida Bay, USA in 1987, provided a unique opportunity to examine the recovery capacity of a subtropical seagrass ecosystem following landscape-scale disturbance (Fig. 1). Notably, this seagrass die-off represented one of the most spatially extensive mortality events of a marine foundation species reported to date^11^.

The geomorphology of Florida Bay, along with substantial modifications to Everglades hydrology, contribute to the hypersaline conditions that promote seagrass die-off. Florida Bay is a shallow lagoon composed of a network of hydrologically discrete basins located at the southern end of the Florida peninsula (Fig. 1 and Supplementary Information A). Because rainfall is the principal source of freshwater delivery to the isolated basins of west-central Florida Bay, basins are prone to hypersalinity during drought^12^. In late summer 1987, high salinities and temperatures in the west-central Bay initiated a cascade of events leading to bottom-water anoxia and sulfide toxicity, resulting in the mass mortality of turtlegrass, the dominant seagrass species (Supplementary Information A). Within months, more than 4000 ha of previously dense turtlegrass meadows, the primary source of benthic structure in the system, were completely denuded, and by 1990, an even larger area was seriously affected^11^ (Fig. 1). When drought conditions precipitating this pulsed event ended in fall 1991, almost a decade of persistent algal blooms and prolonged sediment re-suspension followed, resulting in system-wide reductions in light availability. During the algal bloom period, secondary losses of not only the climax species, turtlegrass, but also all other seagrass taxa were recorded, including the early colonizers *Halodule wrightii* (shoal grass) and *Syringodium filiforme* (manatee grass), effectively halting the beginning stages of successional recovery in locations severely affected by die-off^13^.

An assessment of Florida Bay seagrass communities approximately seven years post die-off revealed little evidence of turtlegrass recovery^13^. Pronouncements of Florida Bay ecosystem collapse and suggestions of regime shifts were advanced^14,15^ and continue to be discussed in the current ecological literature^16^. With few examples of subtropical or tropical seagrass dynamics following disturbance to inform the debate, particularly in response to landscape-scale impacts, the potential for recovery was uncertain. Although data on seagrass (principally *Zostera marina*) recruitment and response to disturbance were available^17–19^, use of models based upon a single species of seagrass from temperate ecosystems proved inadequate because subtropical seagrass assemblages are composed of multiple taxa and different species composition than those in temperate systems. The climax species in Florida Bay, turtlegrass, with a slow growth rate and limited seed production^20,21^, is expected to have a much slower rate of colonization than its successional equivalent in many temperate settings. Consequently, fundamental questions remained regarding mechanisms of recovery, the pattern of successional replacement of taxa and the appropriate time scales over which to assess resilience in Florida Bay.

During the peak of the algal blooms/sediment turbidity (1995), a sampling program (see Materials and Methods) was initiated to quantify ongoing changes in Florida Bay seagrass communities with the scope of the program providing data necessary for a multi-decadal, landscape-scale evaluation of dynamics of seagrass recovery. Four basins located inside the original die-off footprint (Fig. 1) served as target locations. Die-off impacts within basins were characterized as either (1) “severe” or (2) “patchy” (i.e., moderately affected) based upon the pattern of turtlegrass cover observed during the first few years of sampling (Fig. 1; see Materials and Methods). Using this information, two inter-related questions guided our investigation: (1) does evidence support a scenario of seagrass ecosystem recovery in die-off areas relative to historical levels of turtlegrass aboveground biomass (g m^−2^) and/or seagrass cover in undisturbed locations, or (2) are seagrass ecosystem responses post die-off reflective of a regime shift^14^ as evidenced by loss of the foundation species and altered seagrass community structure? Here, we document the time course of foundation seagrass-species recovery in Florida Bay, discuss factors that contribute to ecosystem resilience and explore the long-term implications of our findings.

## Results

### Foundation species response

**Figure 2:**
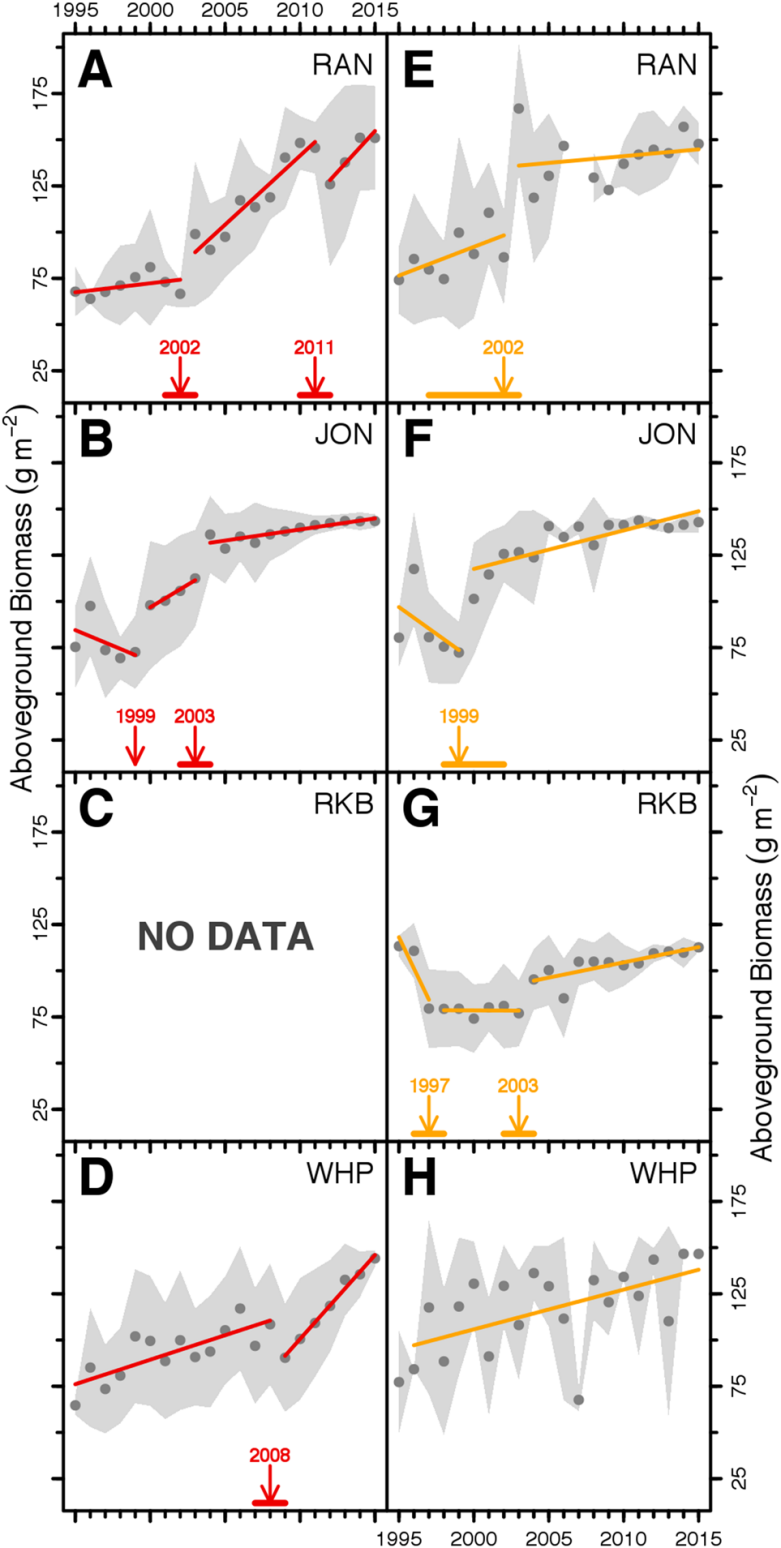
Temporal patterns of seagrass abundance identified by breakpoint analysis. Mean annual aboveground biomass of turtlegrass (circles) is presented for basins within which severe (red; **A**–**D**) and patchy (orange; **E**–**H**) die-off was recorded. Transitions between different phases of recovery were identified using breakpoint analysis (Materials and Methods). Colored horizontal lines along the biomass abscissa indicate 95% confidence intervals for each breakpoint (arrows). Solid lines are linear fits for each segment and standard deviation is shown as the gray shaded region.

**Figure 3:**
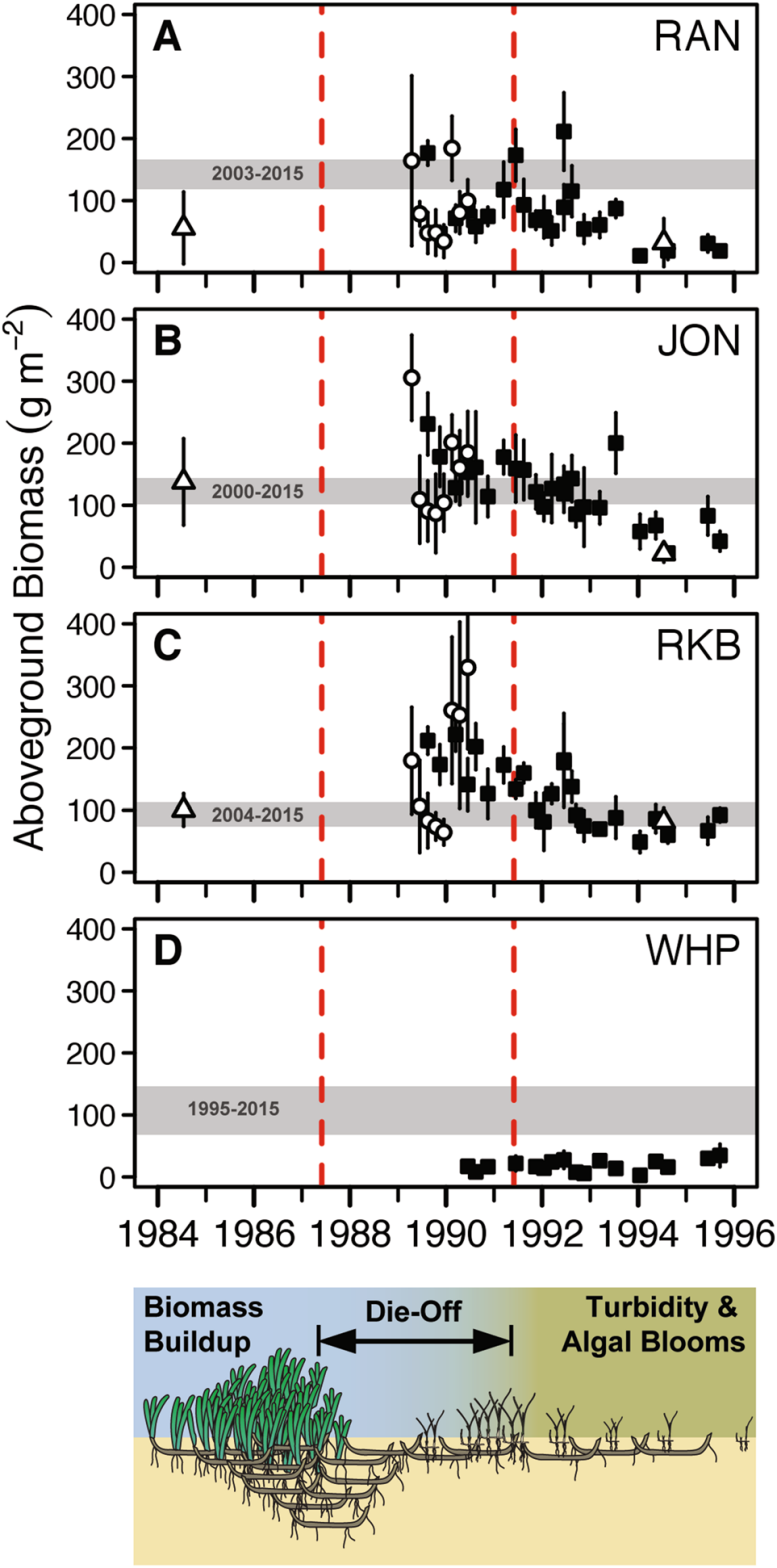
Turtlegrass biomass in die-off zones during the “recovered” phase of post die-off generally met or exceeded pre-die-off levels in all basins. The range in annual mean aboveground biomass of turtlegrass from the last segment of regression of each basin, interpreted as the “recovered” phase, and both die-off zones (shown as gray shaded region and taken from Fig. 2) is compared to unaffected areas. For each basin, years noted within the gray shaded region represent those spanning the last segment identified by breakpoint analyses (Fig. 2). Also presented are historical data for “pre-die-off” biomass of turtlegrass in west-central Florida Bay (open triangle) and values for turtlegrass biomass extracted from studies reporting data for turtlegrass collected from cores within healthy, unaffected turtlegrass beds contemporaneously with die-off (time period within red dashed lines; data sources: squares^51^, triangles^52^, circles^53^). Pre-die-off information for WHP was only available from a nearby location outside sampling boundaries. Decreasing levels of seagrass biomass in unaffected areas from 1991–96 due to algal bloom effects are evident. Sequential changes from 1984–1996 in seagrass response post die-off and prior to recovery are illustrated below plots (see Supplementary Information A).

Sequential changes in seagrass community composition and turtlegrass abundance occurred in all die-off areas surveyed over our 20-y study. Structural Breakpoint Analyses revealed distinct phases of recovery in turtlegrass biomass, one of our target metrics, based on level of impact (“severe” and “patchy”) within the four die-off basins **(**Fig. 2A–H). Rankin Key (RAN) and Johnson Key (JON) Basins, which had highest incidence of severe die-off, exhibited three discrete phases of turtlegrass recovery: (1) a stagnant recovery or depressed phase during post-die-off algal blooms, (2) a recovering phase during which turtlegrass biomass increased, and (3) a recovered phase where turtlegrass biomass was relatively stable over several years. In some cases, seagrass biomass decreased during the first phase [i.e., JON and Rabbit Key (RK) Basins], suggesting bloom-induced secondary mortality (i.e., shoot thinning due to light limitation), but biomass subsequently increased to levels similar to those recorded in the recovered phase of severely affected basins (Fig. 2A–H). Even for severely affected areas, once algal blooms and turbidity subsided, comparatively high turtlegrass biomass was achieved in 5–10 y estimated from breakpoint analyses with three segments (Fig. 2A,B,F). The range of turtlegrass biomass recorded following recovery (Fig. 2A–H) generally met or exceeded levels of turtlegrass abundance present in Florida Bay prior to die-off (Fig. 3A–D). The entire sequence of die-off, algal blooms and recovery took 17–23 y (Figs. 2 and 3).

### Seagrass community structure

**Figure 4:**
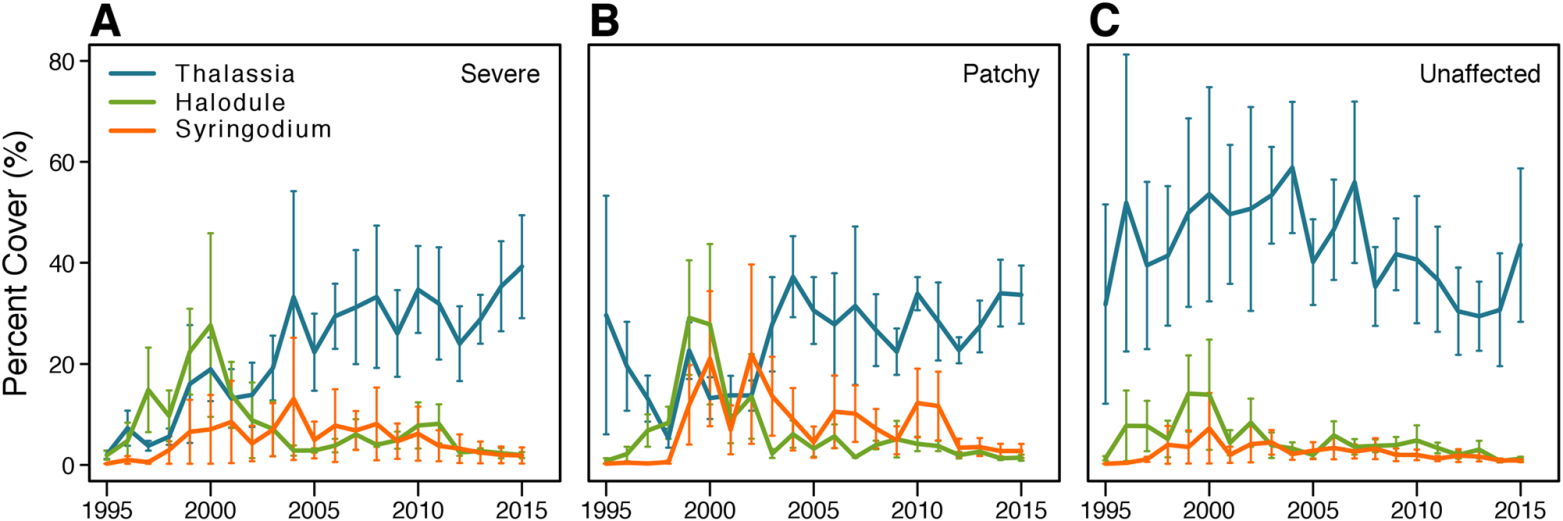
Following die-off, turtlegrass replaced early successional species of seagrass, becoming the dominant canopy former in all basins. Annual mean (± SE) percent cover of three seagrass species recorded over the recovery trajectory by die-off zone: (**A**) severe; n = 3, (**B**) patchy; n = 4, and (**C**) unaffected; n = 4. Basin means (JON, RAN, RKB and WHP) were treated as replicates for each year. Temporal patterns of seagrass coverage in areas of severe die-off exhibited initial colonization by the fast-growing seagrass, shoal grass (*Halodule*), followed by manatee grass (*Syringodium*)*,* coincident with improved light conditions after several years of algal blooms. For patchy zones (**B**), turtlegrass (*Thalassia*) remaining after die-off was lost during the algal blooms. Recovery then exhibited a temporally compressed pattern of the same seagrass succession pathway as in (**A**), above. In contrast, unaffected areas (**C**) retained nominally high levels of cover over the entire 20-y sampling period.

Examination of seagrass community structure confirmed species trajectories reflecting recovery along expected successional pathways, with no ultimate regime shift (Fig. 4A–C). Although a period of algal blooms and sediment turbidity slowed the timeline of recovery, this, too, proved to be a pulsed disturbance. As water clarity improved in the late 1990s and early 2000s^22^ (see also Supplementary Information A), the two fast-growing seagrasses, shoal grass and manatee grass, were the first to respond, forming dense meadows in previously denuded areas. Increases in the slower-growing climax species, turtlegrass, eventually followed (Fig. 4A and B). Over time, seagrass meadows transitioned to the climax community dominated by turtlegrass with respect to areal cover, consistent with the high turtlegrass biomass observed in west-central Florida Bay prior to die-off (Figs. 3, 4A and B). Areas not affected by die-off (Fig. 4C) retained high levels of turtlegrass cover and comparatively low cover of shoal grass and manatee grass over the observation period. Mean percent cover for the three seagrass species in severe and patchy die-off zones displayed similar patterns of recovery (Fig. 4A and B), with turtlegrass becoming the numerically dominant species, and manatee and shoal grass declining to subordinate status by 2003. Thus, in both patchy and severe die-off zones, the climax foundational species regained community dominance 16–17 y after die-off was first observed in 1987 (Fig. 4A and B).

## Discussion

Our results demonstrate that seagrass meadows in Florida Bay dominated by a slow-growing foundation species have the capacity to recover from disturbance, in contrast to earlier advisories that the system was proceeding along a trajectory towards regime shift or ecosystem collapse. Moreover, our findings provide new information on variable rates of recovery among subtropical seagrass taxa over more than two decades post-disturbance (Fig. 4). The sequence and timeline of seagrass species replacement along successional pathways for this subtropical setting during recovery generally agreed with results from small-scale studies of community change in the Caribbean and Gulf of Mexico^23^, and were aligned with outcomes observed in seagrass restoration projects attempting to accelerate succession^24^. However, it is noteworthy that the area over which seagrass succession was observed (40 km^2^) was much larger than previously reported in similar multi-species systems (e.g., 10 to 200,000 m2)^23,25–27^. We also found no evidence that rhizophytic algae were a required first stage of seagrass succession as has been an operating paradigm for subtropical/tropical systems^23^.

Although large-scale seagrass mortality in Florida Bay represented one of the most catastrophic losses of seagrasses on record^28^ with little evidence of seagrass re-establishment for 8–10 y after the first reports of die-off, our long-term observations demonstrated ecosystem recovery within a window of 17–23 y (Figs. 2, 3, 4). We base this conclusion upon a large spatiotemporal dataset, assembled from over 20,000 quadrats sampled across four basins, providing high confidence in our assessment of ecosystem recovery. What is perhaps highly unanticipated is that turtlegrass, the foundation species, re-established dominance without human intervention or the need for reduced human impact, the latter required in 78% of the documented recoveries of marine animal populations/ecosystems^29^. Conceivably, the chronology of recovery could be shortened if restoration efforts were initiated via planting of early colonizing seagrass^24^ after algal blooms ceased, but this is not a likely solution given high costs and logistical difficulties of planting such a large remote area.

### Factors impacting recovery

Previous studies have suggested that the co-occurrence of taxa that vary with respect to growth forms, recruitment potentials and environmental tolerances can enhance ecosystem resilience by improving recovery capacity post-disturbance^30^ and this may be one factor that enhanced seagrass recovery in Florida Bay. The coordinated appearance of multiple seagrass taxa along the recovery trajectory suggests that species richness contributes to resilience at largest spatiotemporal scales. Specifically, subordinate, understory seagrass species rapidly inhabited denuded sediments after light conditions improved (Fig. 4); this may have played a role in facilitating recovery of the foundation species. Potential facilitation mechanisms include reduction of sediment re-suspension events^31^ and mitigation of sediment sulfides in the rhizosphere^32^. Post-disturbance responses by mixed-species seagrass meadows^33^ merit increased attention, as much of our current understanding of seagrass ecosystems globally and discussions of resistance, recovery and/or resilience have been focused on monospecific assemblages in temperate systems^34^.

The capacity for marine ecosystems to recover from disturbance can be impeded by altered trophic interactions and/or competitive outcomes^6^, such as when invasive species assume new roles^35^, or the activities of herbivores alter basal resources^36^. However, none of these mechanisms appeared to operate in Florida Bay. Attached and drift macroalgae, either native or exotic, did not outcompete seagrasses^37^ or negatively affect seagrasses via production of phytotoxins^38^. Likewise, no sustained shifts in the proportional abundance of turtlegrass in the seagrass community were observed following recovery, as might be expected if herbivory rates were altered^39^. In Florida Bay, weak trophic interactions between seagrass and herbivores^40^, may have minimized disruption of food web linkages accompanying the loss of the foundation species^41^, thereby enhancing resilience of the current day seagrass community in Florida Bay.

A set of unique factors may have combined to enhance the recovery capacity of seagrass beds within west-central Florida Bay. First, the die-off was preceded by a discrete period of environmental conditions (i.e., extreme hypersalinity and elevated summer temperatures^32^) and expansion of completely defoliated areas ceased approximately three years after the initiation of die-off, concurrent with the end of the multi-year drought. Second, the location of die-off was in a remote and protected area within Everglades National Park, USA, far removed from areas of dense human population, diminishing the role of anthropogenic effects, such as poor water quality^42^, in initiating or sustaining the event^43^. Similar geographic/environmental features and disturbance mode characteristics have also been linked to resilience and recovery in previous investigations of other marine ecosystems (i.e., frequency of disturbance^4^ and remote conditions^5,7^).

Additionally, light limitation during the algal bloom period did not deplete sources of seagrass recruits. The turtlegrass meadows affected by die-off, while distributed over an extensive area (Fig. 1), were located within a larger constellation of healthy mixed meadows in other portions of Florida Bay, so at the landscape-scale, die-off areas likely maintained connectivity^44^ with unaffected seagrass beds. In locations where die-off and water column turbidity did not lead to complete seagrass loss, remnant patches of turtlegrass and the surviving understory taxa probably served as nodes for clonal expansion. It is likely that the spatial distribution and expansion rate of remnant turtlegrass patches set the tempo for recovery, but only once adverse environmental conditions were abated. Furthermore, flowering of all seagrass species was observed post-disturbance (M.O. Hall, personal observation), and later estimates of turtlegrass clonal diversity and gene flow^45^ appear consistent with sexual recruitment (seedlings) contributing to recovery.

### Implications of study

Our long-term assessment of one of the world’s largest seagrass meadows^46^ provides unprecedented information on seagrass community dynamics and the time scale of recovery following a large-scale disturbance. Given that earlier reports considered the Florida Bay ecosystem to be collapsing^15,47^, our results underscore the challenges with assessing recovery^7^ in locations for which historical information is generally lacking. Our findings that full recovery of the seagrass community, including the foundation species, in west-central Florida Bay required approximately two decades suggest that the time scale over which seagrass ecosystem resilience is currently evaluated, especially at the largest spatial scales, may need to be greatly expanded beyond typical study duration.

Whether mass mortality of turtlegrass in west-central Florida Bay represents a rare event or is a periodic occurrence remains an open question. However, historical accounts of localized turtlegrass die-off^48,49^, regular observations of small die-off patches during annual sampling, and the recurrence of large-scale die-off in 2015 under similar environmental conditions and in the same areas as in 1987–91^50^ collectively indicate that repeated disturbance and the challenge of recovery are common features of the system. If so, the persistence of turtlegrass as the foundation species will likely depend upon the frequency and severity of environmental stressors, poorly documented to date, and their combined impact on the trajectory of seagrass community recovery (i.e., successional processes) and recruitment potential.

Of concern is that future seagrass mortality events in Florida Bay may be exacerbated by elevated sea water temperatures and prolonged droughts associated with climate change, thereby posing unknown challenges to the dynamics of seagrass recovery. Rising sea level leading to increased water exchange among basins and planned management actions to increase freshwater delivery to Florida Bay could mitigate some of the drought-producing conditions associated with die-off events (i.e., hypersalinity). However, our investigation clearly warns that the net influence of natural and new anthropogenic drivers will likely require multi-decadal-scale evaluation to determine whether future seagrass communities will be fundamentally altered, with unknown implications for the provision of ecosystem services, or if turtlegrass can remain the foundation species in west-central Florida Bay.

## Materials and methods

### Experimental design

To investigate our two questions of interest we conducted field sampling of seagrasses in Florida Bay from 1995–2015. Sampling was conducted within four west-central Florida Bay basins (Fig. 1) to collect information on the abundance of three most abundant seagrasses: turtlegrass (*Thalassia testudinum*), the foundation species, as well as subordinate seagrass species (shoal grass; *Halodule wrightii* and manatee grass; *Syringodium filiforme*) over representative areas of the die-off. Voucher specimens for seagrass species were collected and identified by Dr. Michael J. Durako and deposited in the David J. Sieren Herbarium, University of North Carolina at Wilmington. All field activities were conducted under scientific research and collections permits from Everglades National Park (see Supplementary Information B) and were in compliance with relevant institutional, national, and international guidelines and legislation.

#### Basin-wide sampling

Stations from each of four selected basins were sampled using a stratified-random sampling design. All basins were divided into approximately 30 tessellated hexagonal grid cells, and a single station position was randomly chosen from within each grid cell per sampling date (n = 30 per basin). Sampling grids were generated using algorithms developed by the U.S. Environmental Protection Agency (EPA), Environmental Monitoring and Assessment Program (EMAP). At each selected site, seagrass abundance was quantified using a modified Braun-Blanquet technique within eight, randomly chosen, 50-cm × 50-cm quadrats. All seagrass species occurring within the quadrats were assigned a cover/abundance value according to the following scale: 0 = absent; 0.1 = solitary shoots, with small cover; 0.5 = few shoots, with small cover, 1 = numerous shoots, but < 5% cover; 2 = any number of shoots, with 5–25% cover; 3 = any number of shoots, with 26–50% cover; 4 = any number of shoots, with 51–75% cover; 5 = any number of shoots, with 76–100% cover.

#### Permanent transect sampling

Permanent 50-m transects were established in 15 Florida Bay basins in 2006, and seagrass abundance data were collected at 10 random positions along each transect line at the end of the dry (May), and the wet season (October) every year. Specifically, Braun-Blanquet values for all seagrass taxa were determined from within 0.25-m^2^ quadrats and short-shoot densities from 0.1-m^2^ quadrats at each random position. Quantitative measures of seagrass abundance (e.g., aboveground seagrass biomass, short-shoot density) were also determined from within 15-cm diameter PVC cores collected from three haphazardly selected locations adjacent to the transect line on each sampling date. These data were used to convert cover data to above ground biomass (see below).

#### Delineating die-off zones

Our study focused on four basins in west-central Florida Bay substantially affected by the 1987 die-off and algal blooms (JON, RAN, RKB and WHP; Fig. 1). Braun-Blanquet data collected from 1995–2000 were used to delineate zones of “severe” and “patchy” turtlegrass loss within basins based upon two assumptions: (1) meadows affected by die-off were almost totally devoid of turtlegrass and remained so through 2000 (when the algal blooms subsided), and (2), while die-off often resulted in complete turtlegrass loss (i.e., frequency of occurrence near zero), prolonged shading effects (i.e., under algal blooms) were characterized by seagrass shoot thinning and lower cover, but a higher frequency of turtlegrass occurrence than that recorded in die-off areas. To parse these two scenarios, we used mean Braun-Blanquet (BB) scores of turtlegrass cover of ≤ 1 (0 to 5%) and frequency of turtlegrass occurrence of 0.5 as thresholds to define impacts of the die-off as “severe” (BB ≤ 1 and frequency ≤ 0.5) or “patchy” (BB ≤ 1 and frequency > 0.5). Sample locations that did not meet these criteria were designated as “unaffected”. Then, using ArcGIS, 500-m circular buffers were created around each sample location and the number of categories within the circumscribed area per location was tabulated. Using the most common category to describe each buffer, contiguous areas of damage level were merged by hand to form zones of “die-off severity”. In the absence of historical die-off survey data (Supplementary Information A), these methods provided the only sub-basin-level depiction of the spatial distribution of die-off available to guide our sampling design for evaluation of recovery.

### Converting Braun–Blanquet scores to biomass data

Published historical data on Florida Bay turtlegrass abundance, including data collected during the die-off event (Fig. 3), were limited primarily to aboveground biomass estimates derived from benthic cores (15-cm diameter). Because our post-die-off assessment of seagrass abundance from basin-wide sampling (i.e., hexagon sampling) was recorded as Braun-Blanquet data on seagrass species cover, we developed a regression tree model using the package ‘rpart’ to convert BB scores of turtlegrass cover to aboveground biomass (g). The relationship was calculated using permanent transect data for which shoot counts in the random quadrats were matched to shoot counts recovered from biomass cores collected adjacent to transects. Assignment errors were reasonably low, so we could confidently convert basin-wide sampling data into aboveground biomass. One percent of the basin-wide data had to be removed from the predictions step due to Braun-Blanquet score combinations that were not present in the regression tree training data (i.e., transect sampling); this never affected all quadrat samples at any one site.

### Statistical analysis/evaluating recovery

#### Identifying “recovered” biomass levels

We plotted turtlegrass biomass from 1995–2015 for each of the four basins and die-off zones and identified transitions in the trajectories of turtlegrass biomass indicative of recovery using breakpoint analysis via the package ‘strucchange’. This routine works by minimizing Bayesian Information Criterion (BIC) among competing partitions. Breakpoint years (± 95% confidence intervals) were used to delineate transition points along the trajectory. Given the ontogeny of the die-off and secondary algal blooms/turbidity (see schematic Fig. 3), we expected three potential phases of the recovery captured by the biomass dataset: phase 1—turtlegrass biomass static or declining due to lingering impacts of secondary mortality during turbidity and algal bloom period; phase 2—turtlegrass biomass increasing or “recovering” indicative of improving light levels; and phase 3—turtlegrass biomass maintained at high levels or “recovered”. To identify these phases in our biomass data, we used the segments generated by breakpoint analysis. The last phase was of primary interest for detecting time to recovery. When three segments were detected by the breakpoint analysis, we estimated the time required for turtlegrass recovery post-algal bloom/sediment turbidity by tallying the number of years from the end of segment 1 through the first year of segment 3.

#### Comparison of “recovered” biomass to historical biomass levels

Limited historical information exists with which to assess the long-term recovery of Florida Bay turtlegrass communities. Levels of turtlegrass aboveground biomass present prior to die-off were estimated from cores collected in unaffected turtlegrass beds adjacent to die-off patches in JON, RAN and RKB from 1989–1991^51–53^. Turtlegrass recovery was assessed by comparing pre-die-off biomass values to biomass values from the “recovered” phase we identified using breakpoint analyses (Fig. 2). Specifically, a basin was deemed to have recovered if the range in mean annual turtlegrass biomass during the last segment detected by breakpoint analysis (represented by horizontal gray bands in Fig. 3; severe and patchy die-off zones combined) met or exceeded that recorded in unaffected/non die-off areas prior to secondary seagrass losses from high turbidity and algal blooms (Fig. 3; red dashed lines). Historical biomass data from within WHP basin were not available, thus the pre-die-off estimates for WHP were obtained from an area adjacent to current basin boundaries.

#### Community composition in recovered meadows

We also examined the trajectory of seagrass community composition to compare post-die-off assemblages to those of pre-die-off conditions or unaffected areas. We converted Braun-Blanquet data from basin-wide sampling to percent cover for three seagrass species (turtlegrass, shoal grass, and manatee grass) and plotted community trajectories from 1995–2015 using annual mean (± SE) percent cover of seagrass species for all basins by die-off zone (Fig. 4). We judged recovery to have occurred on the date when (1) turtlegrass percent cover, the foundation species, in patchy and severely impacted basins exceeded that of subordinate seagrass species and (2) proportional abundance of seagrass assemblages in impacted basins resembled that from areas unaffected by die-off. Only three seagrass species (turtlegrass, shoal grass, and manatee grass) were sufficiently abundant for this evaluation.

## Supplementary Information

Supplementary Information

## Data Availability

All data related to this paper are on FWC servers and available upon request to B.T.F.
