# Supplementary material for: Natural recovery of a marine foundation species emerges decades after landscape-scale mortality
Source: Sci Rep. 2021 Mar 26;11:6973. doi: 10.1038/s41598-021-86160-y (PMC7997892; doi:10.1038/s41598-021-86160-y)
Supplement: Supplementary file 1 — Supplementary Information [file 41598_2021_86160_MOESM1_ESM.docx]

**Natural recovery of a marine foundation species emerges decades after landscape-scale mortality**

Margaret O. Hall^1^; Susan S. Bell^2^; Bradley T. Furman^1^; Michael J. Durako^3^

^1^Florida Fish and Wildlife Conservation Commission, Florida Fish and Wildlife Research Institute, St. Petersburg, FL 33701

^2^ Department of Integrative Biology, University of South Florida, Tampa FL 33620

^3^ Center for Marine Science, University of North Carolina at Wilmington, Wilmington, NC 28403

**SUPPLEMENTARY INFORMATION:**

**A. Background Information on 1987 seagrass die-off in Florida Bay, USA**

**B. Permits issued for the field work over the study duration**

**(***Citation* *number refers to numbered list of references within main text of article***)**

**A. Background Information on 1987 seagrass die-off in Florida Bay, USA**

Florida Bay is a shallow (1-2 m depth), triangular-shaped estuary located between the Florida mainland and the Florida Keys^54^. Seagrasses represent the dominant benthic macrophytes within Florida Bay^46^ (approximately 2,000 km^2^) and display patterns in density and productivity reflective of gradients in sediment depth and macronutrient availability, all increasing from the northeast to the southwest^52,55^. *Thalassia testudinum* (turtlegrass) is generally the dominant seagrass taxon and canopy former; however, *Halodule wrightii* (shoal grass) and *Syringodium filiforme* (manatee grass) are frequently present, composing mixed meadows with species proportionality related to water quality and successional state^52^. Other species encountered occasionally at our study sites are *Halophila decipiens*, *H. engelmanni* and *Ruppia maritima*.

Despite Florida Bay’s large size, it is a network of distinct basins separated by shallow subtidal-to-intertidal carbonate mud banks, the latter covering nearly 25% of the bay^56,57^. These hydrologically isolated basins are inherently idiosyncratic, and characterized by distinct macrophyte communities, water quality and ecosystem processes^58^. The main external influences on salinity within the bay are exchange with the Gulf of Mexico, atmospheric precipitation, evaporation and terrestrial freshwater input from the Everglades^59^. In general, the bay’s water column is vertically well-mixed and isohaline, however, the complex system of shallow mud banks restricts horizontal advection among basins and with the Gulf of Mexico^60,61^. Thus, Florida Bay’s salinity varies greatly over time and space. In areas with long residence times such as the west-central bay, salinity can rise rapidly to levels twice as high as that of seawater, particularly during drought periods, due to greater evaporation than precipitation and freshwater inflow^12,62^.

Prior to the 1987 die-off in Florida Bay, little information on seagrass abundance or environmental conditions was available for the previously understudied region. The die-off was characterized by rapid and widespread losses of seagrass^11^, affecting approximately 4,000 ha of seagrass meadow between 1987 and 1991 (Fig.1 of main paper) and was first brought to scientists’ attention by fisherman. By 1990, approximately 9,445 ha of turtlegrass were estimated to be severely denuded^11^ (Fig. 1a, main text). The initial die-off was restricted to dense beds of turtlegrass*.* Causal factors have been much debated in the literature^43,46,63,64^; however, a number of manipulative studies demonstrated that environmental drivers (i.e., the single or multiplicative effects of elevated summer temperatures and high spring-summer salinities) resulted in sustained pore- and bottom-water anoxia which can lead to insufficient aeration of roots and rhizomes, toxic levels of sulfide intrusion and plant death^32,65,66^. Freshly-dead biomass would then support further microbial sulfate reduction, increasing porewater sulfide concentrations and expanding (and coalescing) small, isolated die-off patches into ever-larger sizes^65,67^. Implicit in this model is that the presence of large, dense beds of turtlegrass*,* a species with a comparatively high ratio of below to aboveground biomass, is required to initiate and sustain large-scale die-off events^32^. The conditions of large and dense seagrass beds were reportedly widespread in the central and western portions of Florida Bay prior to 1987^52^. As the die-off slowed and the drought ended in 1991, a decade of widespread turbidity due to cyanobacteria-dominated algal blooms and resuspended sediments from loss of seagrass cover led to extensive secondary losses (i.e., thinning) of seagrasses, including shoal grass and manatee grass^67,68^. Water clarity improved gradually as the phytoplankton blooms subsided, presumably as nutrients released by decomposing seagrasses and resuspended sediment were depleted^69^. Eventually light levels were sufficient to support the first phase of seagrass succession (i.e., *Halodule* colonization).

**B. Permits issued for field work over the study duration**

1995 Class A Collecting Permit #930027

1996-1999 Class A Collecting Permit #960091

2000-2002 EVER-2000-SCI-0079

2003-2004 EVER-2003-SCI-0010

2005-2007 EVER-2005-SCI-0038

2008 EVER-2008-SCI-0033

2009-2011 EVER-2009-SCI-0034

2011-2013 EVER-2011-SCI-0042

2013-2015 EVER-2013-SCI-0027

2015-2017 EVER-2015-SCI-0037

References

54 Schomer, N. S. & Drew, R. D. An ecological characterization of the lower Everglades, Florida Bay and the Florida Keys. 246 pp. ( U.S. Fish and Wildlife Service, Office of Biological Services, Washington, D.C., FWS/OBS-82/58.1, 1982).

55 Fourqurean, J. W., Zieman, J. C. & Powell, G. V. N. Phosphorus limitation of primary production in Florida Bay - Evidence from C:N:P ratios of the dominant seagrass *Thalassia testudinum*. *Limnol. Oceanogr.* **37**, 162-171 (1992).

56 Hall, M. O., Madley, K., Durako, M. J., Zieman, J. C. & Robblee, M. B. Florida Bay in *Seagrass Status and Trends in the Northern Gulf of Mexico: 1940-2002* (eds Handley, L., Altsman, D. & DeMay, R.) 243-254 (2007).

57 Peterson, B. J., Chester, C. M., Jochem, F. J. & Fourqurean, J. W. Potential role of sponge communities in controlling phytoplankton blooms in Florida Bay. *Mar. Ecol. Prog. Ser.* **328**, 93-103 (2006).

58 Rudnick, D. T., Ortner, P. B., Browder, J. A. & Davis, S. M. A conceptual ecological model of Florida Bay. *Wetlands* **25**, 870-883, doi:10.1672/0277-5212(2005)025[0870:ACEMOF]2.0.CO;2 (2005).

59 Rudnick, D. T., Chen, Z., Childers, D. L., Boyer, J. N. & Fontaine, T. D. Phosphorus and nitrogen inputs to Florida Bay: The importance of the Everglades watershed. *Estuaries* **22**, 398-416, doi:Doi 10.2307/1353207 (1999).

60 Wang, J. D., Vandekreeke, J., Krishnan, N. & Smith, D. Wind and tide response in Florida Bay. *Bull. Mar. Sci.* **54**, 579-601 (1994).

61 Lee, N. L. *et al.* Circulation and water renewal of Florida Bay, USA. *Bull. Mar. Sci.* **92**, doi:10.5343/bms.2015.1019 (2016).

62 McIvor, C. C., Ley, J. A. & Bjork, R. D. Changes in freshwater inflow from the Everglades to Florida Bay including effects on biota and biotic processes: a review in *Everglades: the Ecosystem and Its Restoration* (eds S. M. Davis & J. C. Ogden) 117-146 (St. Lucie Press, 1994).

63 Durako, M. J. & Kuss, K. M. Effects of *Labyrinthula* infection on the photosynthetic capacity of *Thalassia testudinum*. *Bull. Mar. Sci.* **54**, 727-732 (1994).

64 Lapointe, B. E. & Barile, P. J. Comment on J. C. Zieman, J. W. Fourqurean, and T. A. Frankovich. 1999. Seagrass die-off in Florida Bay: long-term trends in abundance and growth of turtle grass, *Thalassia testudinum*. *Estuaries* **27**, 157-164, doi:10.1007/bf02803569 (2004).

65 Carlson, P. R., Yarbro, L. A. & Barber, T. R. Relationship of sediment sulfide to mortality of *Thalassia testudinum* in Florida Bay. *Bull. Mar. Sci.* **54**, 733-746 (1994).

66 Borum, J. *et al.* The potential role of plant oxygen and sulphide dynamics in die-off events of the tropical seagrass, *Thalassia testudinum*. *J. Ecol.* **93**, 148-158 (2005).

67 Durako, M. J., Zieman, J. C. & Robblee, M. B. in *Florida Bay Science Conference.* (ed W. Nuttle).

68 Durako, M. J., Hall, M. O. & Merello, M. Patterns of change in the seagrass-dominated Florida Bay hydroscape in *Linkages Between Ecosystems in the South Florida Hydroscape: The River of Grass Continues* (eds Porter, J. W. & Porter, K. G.) 515 - 529 (CRC Publ., Boca Raton, FL, 2002).

69 Glibert, P. M. *et al.* Florida Bay: Water quality status and trends, historic and emerging algal bloom problems. *Contrib. Mar. Sci.* **38**, 5-17 (2009).
